# Advanced pancreatic cancer with KRAS wild-type and EGFR-sensitive mutation respond favorably to furmonertinib: A case report

**DOI:** 10.3389/fonc.2023.1151178

**Published:** 2023-04-06

**Authors:** Xiaoting Ma, Xiu Liu, Kai Ou, Manman Zhang, Lizhen Gao, Lin Yang

**Affiliations:** ^1^ Department of Medical Oncology, National Cancer Center/National Clinical Research Center for Cancer/Cancer Hospital, Chinese Academy of Medical Sciences and Peking Union Medical College, Beijing, China; ^2^ Department of Medical Oncology, Beijing Chaoyang Huanxing Cancer Hospital, Beijing, China

**Keywords:** EGFR, furmonertinib, nimotuzumab, pancreatic cancer, targeted therapy

## Abstract

Pancreatic cancer is the leading cause of cancer death, and treatment options are limited and mostly ineffective. The patient we report had an *EGFR* exon 19 deletion and had disease progression in the short term after receiving three front-line treatment regimens. We administered furmonertinib and observed tumor shrinkage, decreased CA19-9. The progression-free survival (PFS) of furmonertinib was 4.7 months, and no adverse effects were observed. However, the patient did not benefit from subsequent nimotuzumab-based therapy. Targeted therapy driven by the detection of genetic signatures in this patient shows potential clinical benefit in refractory advanced pancreatic cancer.

## Introduction

Pancreatic cancer is the seventh most lethal malignant tumor in the world, with a 5-year survival rate of only about 5%. There are about 330000 deaths from this disease worldwide every year (1–3). Even if early-stage pancreatic cancer is surgically removed, its 5-year survival rate is still less than 20% due to distant metastasis and local recurrence. With the application of albumin bound paclitaxel, liposome irinotecan and other chemotherapeutic drugs, the survival time of 50% of patients with metastatic pancreatic cancer has been extended to about 11 months through chemotherapy, but it is far from people’s expectations, and the adverse reactions of patients are serious (4, 5). The occurrence and development of tumors are closely related to gene mutation, signal transduction and microenvironment changes. Currently, targeted drugs targeting vascular endothelial growth factor (VEGF), epidermal growth factor receptor (EGFR), mitogen-activated protein (MEK), fibroblast growth factor receptor (FGFR), phosphatidylinositol-3-kinase (PI3K)/mammalian target of rapamycin (mTOR) pathway and tumor stem cells have shown good efficacy. Therefore, precision therapy based on molecular spectrum analysis has gradually become the treatment direction in the field of pancreatic cancer. This article introduces a case of advanced pancreatic cancer with KRAS wild-type and EGFR sensitive mutation, which showed good effect after receiving furmonertinib.

## Case presentation

**Figure 1:**
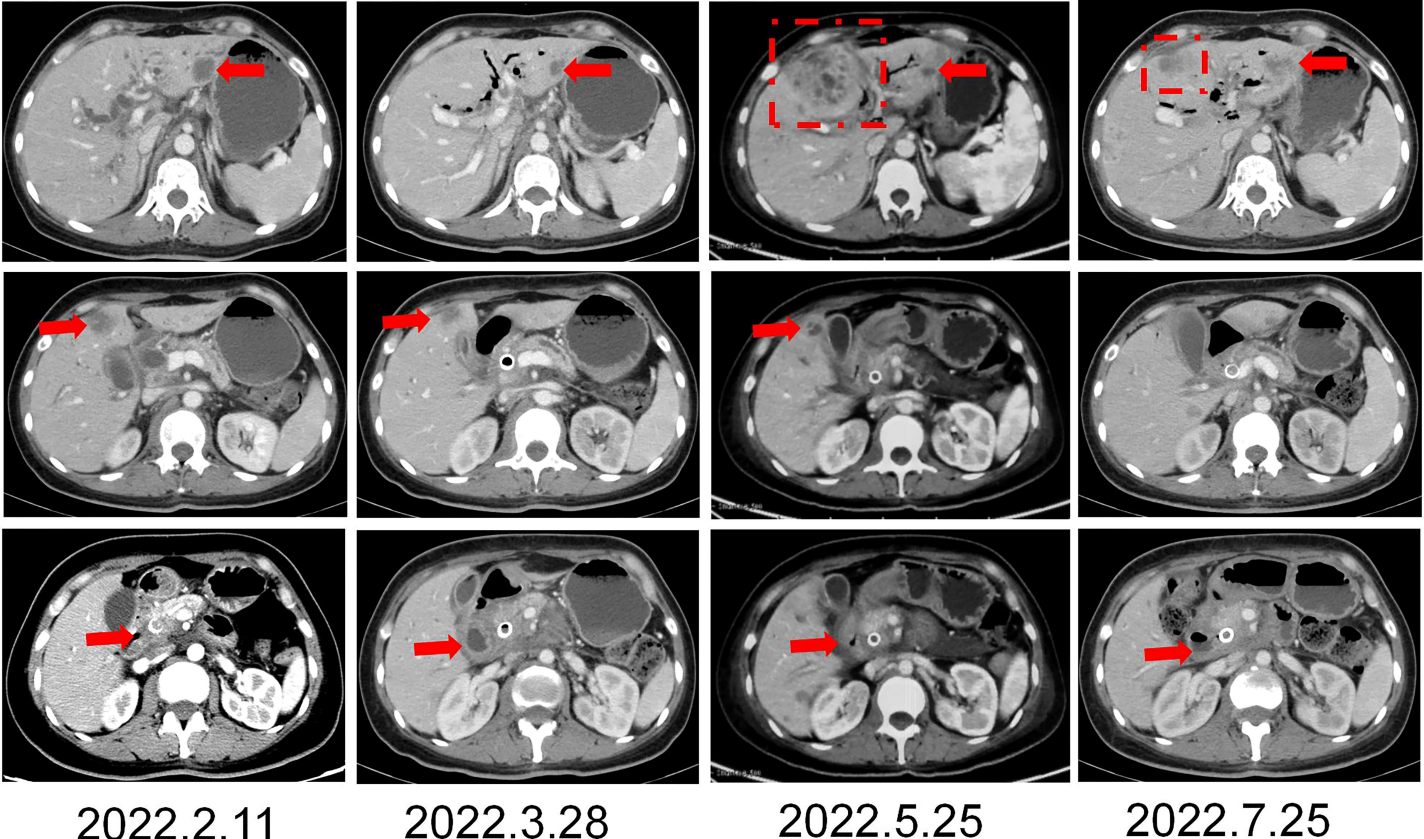
*CT imaging changes in the course of furomertinib treatment.* **(A, B)** CT imaging changes of liver. **(C)** CT imaging changes of pancreas. Arrow: Hepatic metastasis; Dotted line: Hepatic abscess.

**Figure 2:**
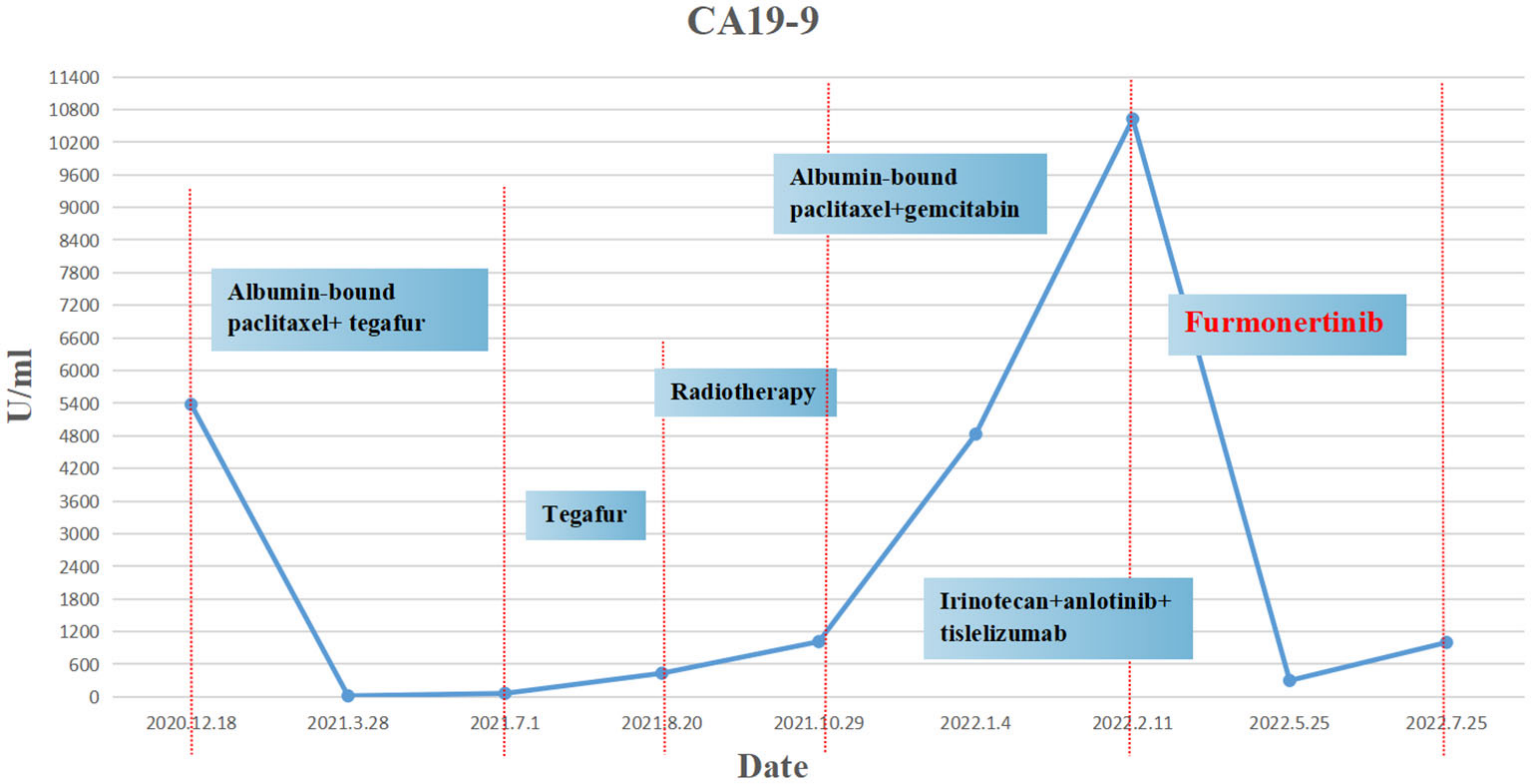
CA19-9 changes throughout the process.

**Figure 3:**
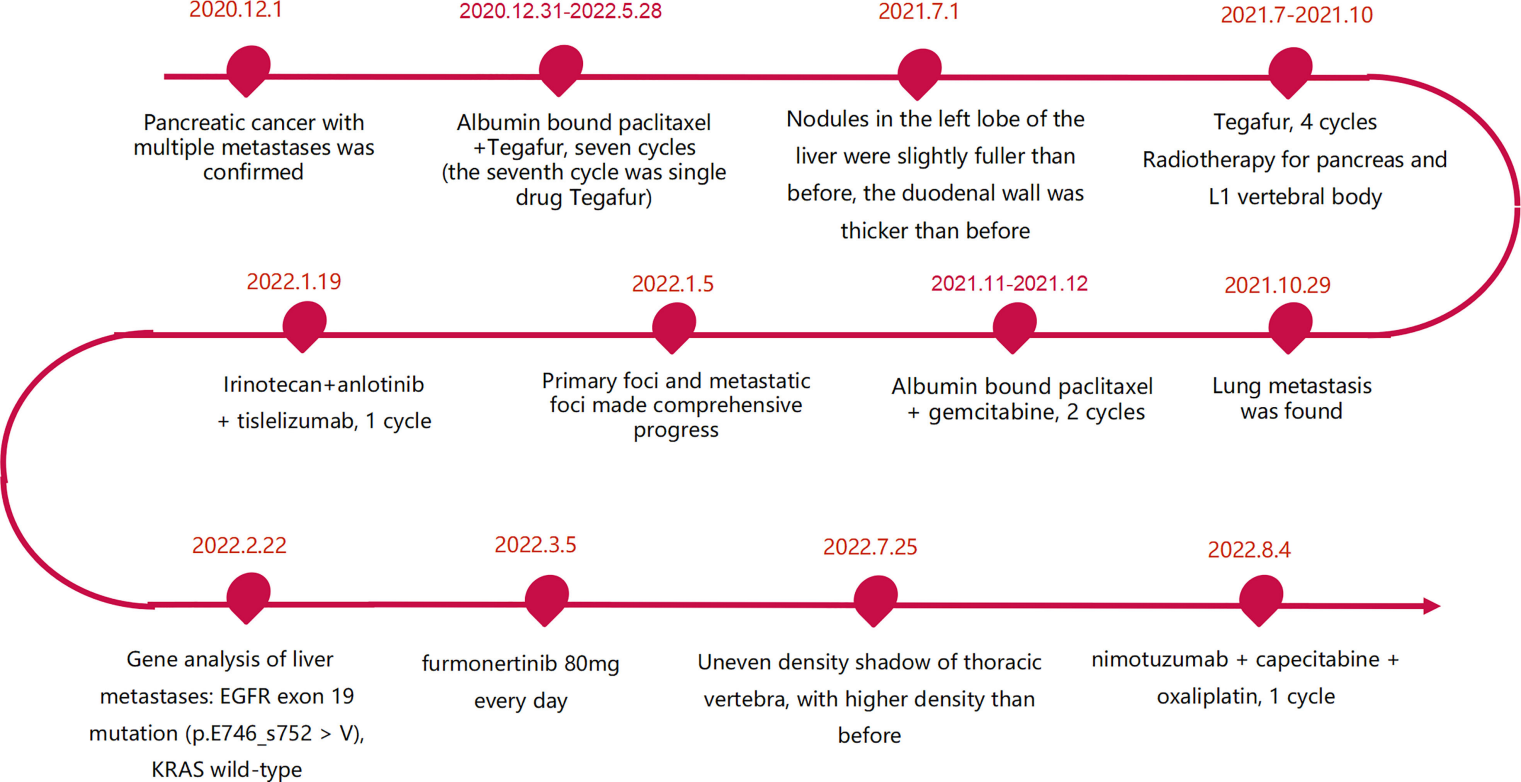
Flow chart of treatment.

The patient was a 45 year old female. In November 2020, she found that his skin and sclera were yellow stained, and went to the local hospital to take the magnetic resonance imaging. It was suggested that the internal and external bile ducts of the liver were expanded in stages, combined with pancreatic duct expansion. The widest diameter of the common bile duct was about 1.3 cm. The computed tomography (CT) examination in our hospital showed that the uncinate process of the head of pancreas was an irregular nodule with unclear boundary and infiltrative appearance, accompanied by low-level biliary obstruction. There are multiple lymph nodes in peripancreatic, retroperitoneal, left gastric region, mesenteric root, hilar region and portal space, and the possibility of metastasis is high. Subcapsular nodules in left inner lobe of liver, nature undetermined, alert for metastasis. Blood test showed that alanine transaminase (ALT): 271 U/L, aspartate transaminase (AST): 154 U/L, total bilirubin (TBIL): 92.8 umol/l, direct bilirubin (DBIL): 78.9 umol/l, indirect bilirubin (IBIL): 13.9 umol/l. On December 1, 2020, encoscopic retrograde cholangio-pancreatography (ERCP), nasobiliary drainage tube implantation and endoscopic ultrasound pancreatic biopsy were performed. Pathology showed adenocarcinoma. On December 22, 2020, ERCP and fully covered metal biliary stent implantation were performed. The postoperative laboratory results showed that ALT: 50 U/L, AST: 28 U/L, TBIL: 22.8 umol/l, DBIL: 14.4 umol/l, IBIL: 8.4 umol/l. From December 31, 2020 to May 28, 2021, seven cycles of albumin bound paclitaxel combined with Tegafur regimen were performed (the seventh cycle was single drug Tegafur). The best efficacy was partial remission (PR), and CA19-9 decreased from 7693 U/ml to 18.09 U/ml. Grade I sensory peripheral neuropathy was found. Bone scanning was taken during the treatment (February 23, 2021), the density of lumbar 1 vertebral body increased, and bone metastasis was considered in combination with CT. On July 1, 2021, CT reexamination showed that the nodules in the left lobe of the liver were slightly fuller than before, and the duodenal wall was thicker than before. Continue to take Tegafur for 4 cycles. 25 fractions of radiotherapy for pancreas and L1 vertebral body were given in the external hospital. On October 29, 2021, the CT reexamination of the hospital suggested multiple small nodules in both lungs, and metastasis was considered. CA19-9 rose to 1015 U/ml. It was evaluated as progressive disease (PD). The patient was treated with albumin bound paclitaxel combined with gemcitabine for 2 cycles at the local hospital. During this period, grade III leukopenia and grade I thrombocytopenia occurred. On January 5, 2022, CT reexamination showed that the irregular soft tissue shadow at the uncinate process of the pancreatic head was larger than before, and the maximum cross-section is now about 3.3 cm × 2.0 cm. There were slightly low-density nodules scattered in the liver, which increased and enlarged compared with the previous ones, and the largest one was about 2.2cm×1.7cm. Metastasis was considered. The lesion of lumbar 1 vertebral body was enlarged. Multiple small nodules were found in both lungs, which were increased and enlarged compared with the previous ones. The largest one is about 0.5cm. Metastasis is considered. On January 11, 2022, CT guided liver tumor puncture was performed. Postoperative pathology suggested that moderately differentiated adenocarcinoma infiltration was seen in the liver tissue. In combination with history and morphology, pancreatic origin was considered. The replacement scheme was irinotecan, anlotinib combined with tislelizumab for 1 cycle. CA19-9 was 10625 U/ml. 2022.2.22 Next-Generation Sequencing result from 520 gene panel of liver metastases showed *EGFR* exon 19 mutation (p.E746_s752 > V), *KRAS* wild-type, tumor mutation burden (TMB) 2 mutations/Mb (Mb stands for every million bases), microsatellite stability (MSS). Immunohistochemistry showed EGFR/ErbB2 2+, ErbB2 1+. On March 5, 2022, she began to take furmonertinib 80mg every day. On May 25, 2022, CA19-9 decreased to 300 U/ml. CT showed the tumor foci of uncinate process of pancreatic head and liver were smaller than before. Liver abscess occurred during the treatment, which gradually subsided after symptomatic treatment. On July 25, 2022, CA19-9 increased to 1000 U/ml. Imaging showed that multiple nodules in both lungs were partially smaller than before, and some were similar to before. Uneven density shadow of thoracic vertebra, with higher density than before, metastasis is considered. The progression-free survival (PFS) of furmonertinib was 4.7 months (**Figures 1**, **2**). On August 2022, the treatment regimen was changed to nimotuzumab, capecitabine combined with oxaliplatin. After 1 cycle, the patient was reexamined due to poor physical condition. CT showed that liver metastases increased and enlarged compared with the previous period, with the largest being about 5.7cm × 3.6cm, suggesting that the effect of nimotuzumab combined with chemotherapy was not good (**Figure 3**). The patient had no previous medical history. The patient’s grandmother had liver cancer and had died, and no genetic tests had been performed.

## Discussion

Pancreatic cancer is the main cause of cancer death, and there is a lack of effective treatment options. Most patients were in the advanced stage at the time of diagnosis, and the regional and distant metastasis rates were 27% and 53%, respectively. It is predicted that pancreatic cancer will become the second leading cause of cancer death by 2030 (6). For many years, chemotherapy has been the cornerstone of the treatment of locally advanced or metastatic pancreatic cancer, and has gradually evolved into an adjuvant or neoadjuvant treatment for resectable pancreatic cancer, but pancreatic cancer shows only moderate sensitivity to chemotherapy (7–9). The occurrence of pancreatic cancer is a complex process involving multiple genes. The related gene mutations mainly occur in *KRAS*, *TP53*, *CDKN2A* and *SMAD4*.

*KRAS* mutation is one of the most lethal drivers of tumor growth and development. More than 90% of pancreatic cancer patients have *KRAS* mutation (10–12). Researchers found that the characteristic mutations of pancreatic cancer are closely related to the precancerous lesions of pancreatic cancer by establishing a model of the occurrence and development of pancreatic cancer (13). *KRAS* mutation was confirmed to occur in almost all levels of precancerous lesions. With the gradual development of precancerous lesions, the frequency of mutation inactivation of *CDKN2A*, *TP53* and *SMAD4* is increasing, which indicates that *KRAS* mutation contributes to its occurrence, and subsequent mutations are important process of tumor progression (14). In recent years, *KRAS* wild-type pancreatic cancer has also attracted people’s attention. These patients account for 10% of the total pancreatic cancer patients (15). With further research, researchers have classified *KRAS* wild-type pancreatic cancer genetic profiling into three types. First, about 4% of *KRAS* wild-type pancreatic cancer is activated by MAPK pathway caused by *BRAF* mutation, and this part of patients can benefit from BRAF inhibitors and MAPK inhibitors. Second, about 2% of *KRAS* wild-type pancreatic cancer has DNA mismatch repair defects and cause microsatellite instability. These tumors often have a high tumor mutation load, can benefit from immunotherapy, and are also sensitive to platinum chemotherapeutic drugs or PARP inhibitors. Third, about 4% of *KRAS* wild-type pancreatic cancer carry some specific kinase gene fusion mutations, such as *ALK*, *NTRK1, NRG1, RET* and *FGFR*, which can benefit from corresponding kinase inhibitors (16).

EGFR is considered to play a role in the pathogenesis of pancreatic cancer. 90% of pancreatic cancer show high expression of EGFR, and high expression of EGFR is usually associated with poor prognosis (17). The high expression of EGFR mainly results from *EGFR* gene amplification or gene mutation. When *EGFR* is amplified, excessive receptors will appear on the cell surface, allowing cells to grow and divide uncontrollably, inducing normal cells to transform into cancer cells, and providing conditions for the sustainable survival of cancer cells. At present, clinical studies on the application of EGFR inhibitors in advanced pancreatic cancer have published the results one after another. Phase III clinical study SWOG 0205 showed that gemcitabine combined with cetuximab failed to improve the survival of patients with advanced pancreatic cancer compared with gemcitabine alone (18). The phase II study in Germany has preliminarily confirmed that patients with high EGFR expression benefit significantly from nimotuzumab treatment, and high EGFR expression is expected to become a new dominant population of nimotuzumab (19). NOTABLE is a prospective, randomized controlled, double-blind, multi-center phase III clinical study in China, which compared the efficacy of nimotuzumab combined with gemcitabine and placebo combined with gemcitabine in the treatment of *KRAS* wild-type locally advanced or metastatic pancreatic cancer. The results showed that nimotuzumab combined with gemcitabine significantly prolonged the overall survival compared with placebo combined with gemcitabine, with median progression-free survival (mPFS) of 4.2 months and 3.6 months, and median overall survival (mOS) of 10.9 months and 8.5 months, respectively. The 1-year survival rates of the two groups were 43.6% and 26.8%, and the 3-year survival rates were 13.9% and 2.7%, respectively (20). This study provides a new therapeutic method for *KRAS* wild-type pancreatic cancer. *EGFR* mutation leads to changes in the behavior of the receptor, causing the receptor to continuously attract epidermal growth factor to the cell surface, which can also promote abnormal cell growth. *EGFR* mutation and *KRAS* mutation are usually mutually exclusive. However, *EGFR* mutation is rare in pancreatic cancer (21–23).

The common mutation sites of *EGFR* occur in exons 18, 19, 20 and 21, of which the non frameshift deletion mutation of exon 19 accounts for about 45%, and the L858R point mutation of exon 21 accounts for 40-45%. These two types of mutations are also known as *EGFR*-sensitive mutations because their response rate to tyrosine kinase inhibitor (TKI) for *EGFR* (EGFR-TKI) exceeds 70%. In June 2022, the third-generation *EGFR* mutant lung cancer targeting drug made in China, furmonertinib, achieved an objective response rate (ORR) of 89% in the treatment of advanced non-small cell lung cancer (NSCLC) and has been approved for first-line treatment of locally advanced or metastatic NSCLC patients with *EGFR* 19 deletion or 21L858R mutation. *EGFR* mutations are most common in NSCLC (24), but *EGFR* mutations are rare in other malignancies. COSMIC database reported *EGFR* mutations in 6 of 1599 PDACs (0.37%) (23). In this case, we found that the patient had an *EGFR* exon 19 deletion through genetic testing. It is a sensitive mutation site of EGFR-TKI that has been marketed. In the field of pancreatic cancer, there are few studies on EGFR inhibitors. Moore et al. performed a phase III clinical study of pancreatic cancer patients, but did not distinguish the status of *KRAS*. The results confirmed that the addition of the EGFR inhibitor erlotinib to gemcitabine could bring a statistically significant advantage over gemcitabine alone, with mOS of 6.3 months and 5.9 months, respectively (25). Although the extended time is limited, this may be related to the indistinguishable *KRAS* status. Driver genes are important genes involved in the development of cancer and previous study has shown that using precision medicine can have a significant effect on survival in patients with pancreatic cancer (26). Another previous case report from our group also reported the effectiveness of precision medicine in patients with pancreatic cancer (27). Considering that this patient had *EGFR* exon 19 deletion, we gave the patient furmonertinib, observed tumor shrinkage, decreased CA19-9, and obtained PFS for 4.7 months, during which no adverse reactions were observed. The patient had received third-line treatment before, and his condition progressed after applying a variety of chemotherapy drugs and immune checkpoint inhibitors. Since then, with the emergence of NOTABLE results (20), we have continued to try to use the treatment based on nimotuzumab, but the patients have not benefited from this treatment. This may suggest that EGFR2+ patients may not benefit from nimotuzumab. However, since we failed to perform another pathological test on this patient after furmonertinib resistance, it is not known whether the patient has other secondary mutations. As of September 2022, the OS has been followed up for more than 21 months.

This article is based on the case report of a single case, so it cannot be used as the standard treatment plan for such patients, but it can also provide treatment ideas for this special population. Although the patient’s PFS with furmonertinib were not long, tumor shrinkage was observed on imaging, confirming the effectiveness of this regimen. But we also need to think further about the possible mechanisms underlying furmonertinib resistance. In the field of NSCLC, third-generation EGFR-TKI has significant efficacy, but acquired resistance to tumor development is unavoidable. The related contents involving on - target drug resistance and off - target drug resistance have been reported (28). In addition, current study has suggested that pancreatic cancer has a high degree of intratumor heterogeneity, which is also a major obstacle to effective treatment of pancreatic cancer (29). In the future, a variety of tests, including liquid biopsies, may help overcome the treatment bottleneck for refractory tumors.

## Conclusions

At present, due to the lack of tumors in biological samples and the low incidence of operable genomic lesions, the widespread use of precision medicine in pancreatic cancer patients is limited. However, recent experience shows that genomic analysis is feasible in most patients with pancreatic cancer, and it is possible to obtain mutation features with clinical guiding significance. This case showed potential clinical benefits in refractory advanced pancreatic cancer through targeted therapy driven by detected gene characteristics.

## Data availability statement

The original contributions presented in the study are included in the article/supplementary material. Further inquiries can be directed to the corresponding author.

## Ethics statement

The studies involving human participants were reviewed and approved by Departments of Ethics Committee, National Cancer Center/National Clinical Research Center for Cancer/Cancer Hospital, Chinese Academy of Medical Sciences and Peking Union Medical College. The patients/participants provided their written informed consent to participate in this study.

## Author contributions

XM designed the article form and wrote the manuscript. XL and KO consulted and browsed the literature. MZ and LG collected and followed up patient information. LY revised the manuscript. All authors contributed to the article and approved the submitted version.
